# Priming against environmental challenges and proteomics in plants: Update and agricultural perspectives

**DOI:** 10.3389/fpls.2012.00216

**Published:** 2012-09-11

**Authors:** Georgia Tanou, Vasileios Fotopoulos, Athanassios Molassiotis

**Affiliations:** ^1^ Department of Biochemistry and Biotechnology, University of ThessalyLarissa, Greece; ^2^ School of Agriculture, Aristotle University of ThessalonikiThessaloniki, Greece; ^3^ Department of Agricultural Sciences, Biotechnology and Food Science, Cyprus University of TechnologyLimassol, Cyprus

**Keywords:** plants, priming, proteomics, acclimation, abiotic stress

## Abstract

Priming is the cellular state in which the harmful effects of abiotic stress factors in plants are hindered by pre-exposure to a stimulus, thus resulting in greater survival. It is becoming increasingly evident that priming techniques (e.g., external application of natural or synthetic compounds in plants) can enhance the tolerance of crops to environmental stresses. Innovative systems biology approaches such as proteomics are currently recognized as essential tools to understand the molecular mechanisms underlying plant responses to environmental stimuli and priming phenomena. The few published proteomic studies on priming in the context of environmental stress identify key protein targets and signaling pathways which are being involved in the alleviation of negative effects of stress factors. Since priming is a very promising strategy in modern crop production management, further research is needed in order to establish the global picture of priming phenomena against environmental challenges as well as to characterize specific priming-related protein indicators in plants.

## INTRODUCTION

**FIGURE 1:**
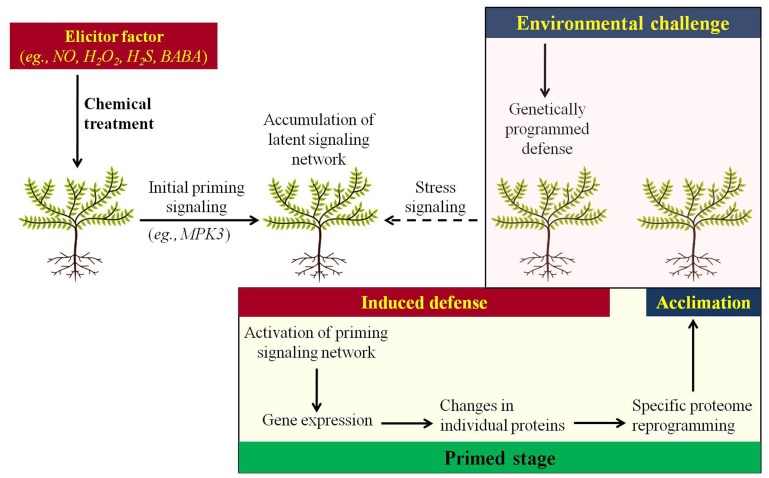
**Model describing the priming-based plant acclimation to environmental challenges.** Chemical treatments of plants with priming elicitors resulted in the initial priming signaling leading the plants to a primed stage, in which a complex priming-related signaling network still remains inactive. Exposure to environmental stress stimulates the genetically programmed defense response in plants together with the activation of priming-related signaling network. This priming framework ensures spatiotemporally appropriate patterns of downstream stress-related gene and protein expression, ending in a specific proteome reprogramming that ultimately leads to stress acclimation.

The process of priming involves prior exposure to an eliciting factor making plants more tolerant to future stress exposure (**Figure 1**). Since environmental stress negatively affects crop growth and productivity throughout the world (Krasensky and Jonak, 2012), studies on plant priming against abiotic stress factors are essential. Despite the agronomic and ecological importance of priming, however, little is known about the molecular mechanisms of priming in plants (Conrath, 2011). Meanwhile, proteomics is becoming a powerful tool to analyze the protein networks in plants upon imposition of environmental stimuli (Hossain et al., 2012). A number of recent studies also pointed out the role of protein post-translational modifications (PTMs), notably carbonylation and *S*-nitrosylation in plant defense and priming (Tanou et al., 2009, 2012; Astier et al., 2012), indicating that redox proteomics is critical in the study of priming responses. Thus, it is apparent that primed plants provide excellent scientific challenges for proteomic analyses which, among others, could significantly improve our understanding of how plant cells effectively respond to environmental cues. The aim of this mini-review is to provide an up-to-date overview demonstrating proteomic approaches used to characterize priming phenomena in plants toward environmental challenges.

## PRIMING PHENOMENA IN PLANTS

Priming is an important mechanism of various induced resistance phenomena in plants against biotic stresses (Beckers and Conrath, 2007), whereas an analogy exists for vaccinated animals for an adaptive immunity to a disease and the ultimate prevention or amelioration of the pathogens infection effects. Proposed priming mechanisms include the accumulation of signaling proteins or transcription factors in an inactive form or the occurrence of epigenetic changes that are modulated upon exposure to stress and developed rapidly resulting in a more efficient defense mechanism (Bruce et al., 2007). Over the past few years, it has become apparent that priming phenomena are also involved in the context of environmental stress (Filippou et al., 2012). Several studies have examined priming events against environmental stimuli in various plant systems. For example, this was evidenced in the case of NaCl pre-treatment on *Glycine max* seedlings in order to induce acclimation to subsequent salt stress (Umezawa et al., 2000), acclimation of *Deschampsia antarctica* to cold stress (Chew et al., 2012) or application of low levels of Cd to *Triticum aestivum* for consequent Cd toxicity acclimation (Li and Zhou, 2012). Similar findings were shown for polyethylene glycol pre-treatment of *Elaeagnus oxycarpa* seedlings in order to induce acclimation to salinity (Murata et al., 2012), drought preconditioning of *Lolium perenne* for cold acclimation (Hoffman et al., 2012), and application of low levels of Zn for subsequent Cd toxicity acclimation in wheat (Li and Zhou, 2012). More interestingly, a primed state could also be induced in plants following an initial exposure to a priming agent, such as natural or synthetic compounds including nitric oxide (NO; Molassiotis et al., 2010), hydrogen peroxide (H_2_O_2_; Molassiotis and Fotopoulos, 2011), hydrogen sulfide (H_2_S; Li et al., 2012), β-aminobutyric acid (BABA; Tsai et al., 2011; Ton et al., 2005), and polyamines (Alcázar et al., 2010; **Figure 1**). This chemical-based priming against abiotic stresses somewhat resembles the systemic acquired resistance phenomenon in the case of biotic stresses (Stuiver et al., 1992; Uchida et al., 2002; Yang et al., 2007). Additionally, in analogy to the priming treatments on the vegetative parts of plants, seed water-based priming with controlled imbibition for seed invigoration and advance in germination, in which long lasting effects occur after germination as well, has been widely characterized (Gallardo et al., 2001). This experimental evidence indicates that priming against environmental stress represents a fruitful area for future research in terms of both basic and applied agriculture science in order to promote the advent of, the more environmentally friendly, sustainable agriculture.

## PROTEOMICS AND PRIMING

The agricultural relevance of priming in plants, as it is a cost-efficient strategy that increases the plant’s ability to cope with stress, has motivated scientists to unravel the underlying cellular mechanisms (Beckers and Conrath, 2007). The last years studies using transcriptome and metabolome techniques have been conducted identifying transcriptional regulators and metabolite switchers and providing fundamental clues of how different networks are affected and interact during the priming process (Zimmerli et al., 2008; Luo et al., 2009). However, constraints in the estimation of gene expression levels, mRNA degradation or inefficiently translation, gene alternative splicing, as well as protein PTMs, processing and protein turnover, make the use of proteomics an essential tool covering the gap between the transcriptome and the metabolome (Renaut et al., 2006). In addition, proteomics studies give the opportunity to track subcellular proteomes and protein complexes (e.g., proteins in the plasma membranes, chloroplasts, mitochondria, and nuclei) and most importantly PTMs associated with priming (see later). Following two dimensional protein extract separation, together with the latest advances in mass spectrometry (MS)-based proteomics such as ion mobility separations, microchip-based proteome measurements, nanoscale reversed phase liquid chromatography, and capillary electrophoresis, as recently described by Angel et al. (2012), fundamental solutions exempting researchers from the elaborate protein separation procedure are now provided. Furthermore, significant developments in protein identification technologies (MALDI-TOF) and quantification strategies such as difference gel electrophoresis (DiGE), together with the availability of web accessible protein databases and the amount of information which are constantly being obtained through full sequencing of major agricultural plant species genomes (e.g., http://www.nature.com/nature/journal/v485/n7400/abs/nature11119.html#supplementary-information), continuously improve the efficiency and reliability of proteomics rendering them a major field of functional genomics.

During the last years, various proteomic strategies characterize the protein changes in primed plants upon environmentally stressful conditions. Using citrus plants exposed to salinity, it was shown that H_2_O_2_ and sodium nitroprusside (SNP, a NO donor) were able to prevent the accumulation of a large number of NaCl-responsive proteins via specific proteome reprogramming (**Figure 1**), which could prepare the citrus plant to respond more effectively to salt stress (Tanou et al., 2009). Furthermore, Bai et al. (2011), using a different NO donor (*S*-nitroso-*N*-acetylpenicillamine, SNAP), showed that the accumulation of G-protein-associated proteins and the induction of antioxidant enzymes were the master mechanism through which salt alleviation was achieved in maize seedlings, in addition to activation of defense proteins, energy metabolism, and cell structure/division remodeling. Notably, the overlapping and/or unique routes of activated mechanisms among different priming agents was identified in BABA- or ABA-treated crabapple plants under drought conditions (MacArisin et al., 2009), as well as in H_2_O_2_- or NO-treated plants upon salinity (Tanou et al., 2009). In specific, the similarities that were identified by MacArisin et al. (2009) in the proteome of ABA- and BABA-treated crabapple seedlings using DiGE showed that BABA-induced abiotic stress tolerance is achieved by potentiating an ABA-regulated pathway. However, the significant differences in proteomic pattern between ABA- and BABA-treated crabapple seedlings indicate that BABA may also mediate drought tolerance via some ABA-independent pathways, including changes in cell-wall enzymes leading to suppression of lignin biosynthesis (MacArisin et al., 2009). Furthermore, Tanou et al. (2009) showed that H_2_O_2_- and NO-originated priming against salinity in citrus plants exhibit a number of similarities in terms of proteome expression signatures, as 45 leaf proteins were commonly targeted by H_2_O_2_ and NO during acclimation to salt stress. It is noted that the majority of H_2_O_2_- and NO-responsive proteins in this study corresponded to enzymes involved in Calvin cycle, such as 1,6-bisphosphate aldolase, phosphoglycerate kinase, glyceraldehyde-3-phosphate dehydrogenase, phosphoribulokinase, transketolase, and carbonic anhydrase, suggesting that reprogramming of specific pathways is required to achieve efficient priming-driven tolerance against salinity. In the same study, both H_2_O_2_ and NO pre-treatments alleviated salinity-induced protein carbonylation and shifted the accumulation levels of leaf *S*-nitrosylated proteins to those of unstressed control plants, suggesting that the oxidation and *S*-nitrosylation patterns of leaf proteins are specific molecular signatures of citrus plant vigor under stressful conditions (Tanou et al., 2009).

One of the best-characterized priming effects in biological systems concerns the application of pre-germination treatments in order to synchronize seed germination and invigorate the produced seedlings against adverse environments (Heydecker et al., 1973; Bradford, 1986; Chojnowski et al., 1997; Harris et al., 1999). The relevance of proteomics to characterize the mechanisms of seed imbibition and priming is best exemplified by studies showing that early steps of seed germination do not require *de novo* transcription while protein synthesis from the mRNAs stored in the mature seeds is absolutely required, hence revealing the role of proteins stored in the dry mature seed or translated from the stored mRNAs in the success of seed germination and seedling establishment (Gallardo et al., 2004; He et al., 2011). Early studies were also focused on the proteome characterization of model plants like *Arabidopsis* under the germinating developmental stages (Gallardo et al., 2001), followed by research on potential markers of seed vigor under primed and non-primed conditions in agricultural species such as alfalfa and sugar beet (Catusse et al., 2011; Yacoubi et al., 2011). Rajjou et al. (2006) proposed salicylic acid (SA), an elicitor of plant defense treatment as an invigorating application promoting *Arabidopsis* seed germination under saline conditions. SA re-induced the late maturation program during early stages of germination, affected the quality of protein translation, primed seed metabolism, provoked the synthesis of antioxidant enzymes, and mobilized seed storage proteins, as evidenced by a proteome-wide analysis (Rajjou et al., 2006). Interestingly, the oxyproteome of *Arabidopsis* seeds was remarkably affected by SA (Rajjou et al., 2006), whereas protein oxidation and seed dormancy alleviation was also achieved in subsequent studies via seed pre-germination treatments with oxidizing compounds like methyl viologen (Oracz et al., 2007) or H_2_O_2_ (Barba-Espín et al., 2011). Similarly, protein carbonylation levels were strongly suppressed in salt-stressed citrus plants experiencing priming phenomena via treatments with H_2_O_2_ or NO before salt stress (Tanou et al., 2009). Using the same experimental system as well as a comprehensive proteomic analysis, the latter further showed that *S*-nitrosylation, the covalent attachment of an NO group to a reactive Cys thiol to form an *S*-nitrosothiol (SNO), as well as tyrosine nitration, the addition of a nitro group (NO_2_) to one of the two equivalent ortho-carbons of the aromatic ring of tyrosine (Tyr) residues, are both involved in the acclimation of citrus plants to salinity conditions (Tanou et al., 2012). These observations indicate that redox proteomic approaches, like protein oxidation (carbonylation), nitrosylation, and nitration represent an important tool toward understanding priming phenomena in plants. Besides proteomic approaches, individual proteins have also been characterized as key components in the priming process. For example, a reverse genetics approach in *Arabidopsis* revealed that chemical priming is based on enhanced accumulation of mitogen-activated protein kinase 3 (MPK3) upon exposure to biotic or abiotic stresses (Beckers et al., 2009), suggesting MPK3 as a potential candidate for priming signaling (**Figure 1**).

## FUTURE PERSPECTIVES ON PRIMING PROTEOMICS

It is noteworthy that several priming-induced chemical compounds, such as NO are increasingly recognized as mobile elements within plants (Molassiotis et al., 2010). Thus, because NO acts on proteins to alter their function and on the metabolic or signaling pathways in which these proteins are involved, it is important to characterize the systemic nature of priming phenomena in plants using proteomic approaches. In addition, many priming studies in plants use model chemical pre-treatment systems to induce priming against abiotic stress. This observation possibly indicates that primed plants possess molecular mechanisms that allow them to memorize previous priming events and generate memory imprints during the establishment of priming (Conrath, 2006, 2011; Bruce et al., 2007; Jaskiewicz et al., 2011; Slaughter et al., 2012; Tanou et al., 2012). Hence, a great deal of additional work is required to understand the systemic as well as the “memory” characteristics of priming and responsible protein-associated mechanisms in plants, especially in the era of redox-based PTMs. In addition, it has been hypothesized that priming involves accumulation of latent signaling components that are not used until challenge by exposure to stress (Beckers et al., 2009). It is therefore interesting to apply comparative proteomics analysis in plants treated with chemical priming agents and before the imposition of abiotic stress conditions. Since seed priming and plant priming involved different mechanisms it would be important to characterize distinct features as well as the potential interplay between them at proteome level. Finally, an intriguing challenge will be the application of the classic idea of priming in various biological processes with important agronomic features. For example, Minas et al. (2012) using a proteomic analysis showed that prolonged ozone application during cold storage in kiwifruits may act as antioxidant priming agent and anti-radical elicitor capable of rendering fruits more tolerant to subsequent senescence and ripening at room temperature. Clearly, the employment of proteomic approaches in priming agent-induced acclimation of plants to environmental challenges represents one of the most promising areas of fundamental and applied research for several years to come, whereas future proteomic studies on seed priming will also help to reveal novel markers of seed quality useful to ensure the best crop yields (Catusse et al., 2011; Corbineau, 2012).

## Conflict of Interest Statement

The authors declare that the research was conducted in the absence of any commercial or financial relationships that could be construed as a potential conflict of interest.

## References

[B1] Alcázar R. Altabella T. Marco F. Bortolotti C. Reymond M. Koncz C. Carrasco P. Tiburcio A. F. (2010). Polyamines: molecules with regulatory functions in plant abiotic stress tolerance. *Plant* 231 1237–1249 10.1007/s00425-010-1130-0 20221631

[B2] Angel T. E. Aryal U. K. Hengel S. M. Baker E. S. Kelly R. T. Robinson E. W. Smith R. D. (2012). Mass spectrometry-based proteomics: existing capabilities and future direction. *Chem. Soc. Rev.* 41 3912–3928 22498958 10.1039/c2cs15331a PMC3375054

[B3] Astier J. Kulik A. Koen E. Besson-Bard A. Bourque S. Jeandroz S. Wendehenne O. D. (2012). Protein *S*-nitrosylation: what’s going on in plants? *Free Radic. Biol. Med.* 53 1101–1110 22750205 10.1016/j.freeradbiomed.2012.06.032

[B4] Bai X. Yang L. Yang Y. Ahmad P. Yang Y. Hu X. (2011). Deciphering the protective role of nitric oxide against salt stress at the physiological and proteomic levels in maize. *J. Proteome Res.* 10 4349–4364 21846115 10.1021/pr200333f

[B5] Barba-Espín G. Diaz-Vivancos P. Job D. Belghazi M. Job C. Hernández J. A. (2011). Understanding the role of H_2_O_2_ during pea seed germination: a combined proteomic and hormone profiling approach. *Plant Cell Environ.* 34 1907–1919 21711356 10.1111/j.1365-3040.2011.02386.x

[B6] Beckers G. J. M. Conrath U. (2007). Priming for stress resistance: from the lab to the field. *Curr. Opin. Plant Biol.* 10 1–7 17644024 10.1016/j.pbi.2007.06.002

[B7] Beckers G. J. M. Jaskiewicz M. Liu Y. Underwood W. R. He S. Y. Zhang S. Conrath U. (2009). Mitogen-activated protein kinases 3 and 6 are required for full priming of stress responses in *Arabidopsis thaliana*. *Plant Cell* 21 944–953 19318610 10.1105/tpc.108.062158 PMC2671697

[B8] Bradford K. J. (1986). Manipulation of seed water relations via osmotic priming to improve germination under stress conditions. *HortScience* 21 1105–1112

[B9] Bruce T. J. A. Matthes M. C. Napier J. A. Pickett J. A. (2007). Stressful “memories” of plants: evidence and possible mechanisms. *Plant Sci.* 173 603–608

[B10] Catusse J. Meinhard J. Job C. Strub J. Fischer U. Pestsova E. Westhoff P. Van Dorsselaer A. Job D. (2011). Proteomics reveals potential biomarkers of seed vigor in sugarbeet. *Proteomics* 11 1569–1580 21432998 10.1002/pmic.201000586

[B11] Chew O. Lelean S. John U. P. Spangenberg G. C. (2012). Cold acclimation induces rapid and dynamic changes in freeze tolerance mechanisms in the cryophile *Deschampsia antarctica* E. Desv.* Plant Cell Environ.* 35 829–837 10.1111/j.1365-3040.2011.02456.x 22070607

[B12] Chojnowski M. Corbineau F. Come D. (1997). Physiological and biochemical changes induced in sunflower seeds by osmopriming and subsequent drying, storage and aging. *Seed Sci. Res.* 7 323–331

[B13] Conrath U. (2006). Systemic acquired resistance. *Plant Signal. Behav.* 1 179–184 19521483 10.4161/psb.1.4.3221 PMC2634024

[B14] Conrath U. (2011). Molecular aspects of defence priming. *Trends Plant Sci.* 16 524–531 21782492 10.1016/j.tplants.2011.06.004

[B15] Corbineau F. (2012). Markers of seed quality: from present to future. *Seed Sci. Res.* 22 S61–S68

[B16] Filippou P. Tanou G. Molassiotis A. Fotopoulos V. (2012). “Plant acclimation to environmental stress using priming agents,” in *Plant Acclimation to Environmental Stress*eds Tuteja N. Gill S. S. (New York: Springer Science and Business Media) (in press). ISBN 978-1-4614-5000-9

[B17] Gallardo K. Debeaujon I. Vandekerckhove J. Job C. Job D. (2004). The effect of α-amanitin on the *Arabidopsis* seed proteome highlights the distinct roles of stored and neosynthesized mRNAs during germination. *Plant Physiol.* 134 1598–1613 15047896 10.1104/pp.103.036293 PMC419834

[B18] Gallardo K. Job C. Groot S. P. C. Puype M. Demol H. Vandekerckhove J. Job D. (2001). Proteomic analysis of *Arabidopsis* seed germination and priming. *Plant Physiol.* 126 835–848 11402211 10.1104/pp.126.2.835 PMC111173

[B19] Harris D. Joshi A. Khan P. A. Gothkar P. Sodhi P. S. (1999). On-farm seed priming in semiarid agriculture: development and evaluation in maize, rice and chickpea in India using participatory methods. *Exp. Agric.* 35 15–29

[B20] He D. Han C. Yao J. Shen S. Yang P. (2011). Constructing the metabolic and regulatory pathways in germinating rice seeds through proteomic approach. *Proteomics* 11 2693–2713 21630451 10.1002/pmic.201000598

[B21] Heydecker W. Higgins J. Gulliver R. L. (1973). Accelerated germination by osmotic seed treatment. *Nature* 246 42–44

[B22] Hoffman L. DaCosta M. Ebdon J. S. Zhao J. (2012). Effects of drought preconditioning on freezing tolerance of perennial ryegrass. *Environ. Exp. Bot.* 79 11–20

[B23] Hossain Z. Nouri M. Z. Komatsu S. (2012). Plant cell organelle proteomics in response to abiotic stress. *J. Proteome Res.* 11 37–48 22029473 10.1021/pr200863r

[B24] Jaskiewicz M. Conrath U. Peterhänsel C. (2011). Chromatin modification acts as a memory for systemic acquired resistance in the plant stress response. *EMBO Rep.* 12 50–55 21132017 10.1038/embor.2010.186 PMC3024125

[B25] Krasensky J. Jonak C. (2012). Drought, salt, and temperature stress-induced metabolic rearrangements and regulatory networks. *J. Exp. Bot.* 63 1593–1608 22291134 10.1093/jxb/err460 PMC4359903

[B26] Li D. D. Zhou D. M. (2012). Acclimation of wheat to low-level cadmium or zinc generates its resistance to cadmium toxicity. *Ecotoxicol. Environ. Saf.* 79 264–271 22284823 10.1016/j.ecoenv.2012.01.012

[B27] Li L. Wang Y. Shen W. (2012). Roles of hydrogen sulfide and nitric oxide in the alleviation of cadmium-induced oxidative damage in alfalfa seedling roots. *Biometals* 25 617–631 22538639 10.1007/s10534-012-9551-9

[B28] Luo Z. Janz D. Jiang X. Göbel C. Wildhagen H. Tan Y. Rennenberg H. Feussner I. Polle A. (2009). Upgrading root physiology for stress tolerance by ectomycorrhizas: insights from metabolite and transcriptional profiling into reprogramming for stress anticipation. *Plant Physiol.* 151 1902–1917 19812185 10.1104/pp.109.143735 PMC2785981

[B29] MacArisin D. Wisniewski M. E. Bassett C. Thannhauser T. W. (2009). Proteomic analysis of β-aminobutyric acid priming and abscisic acid – induction of drought resistance in crabapple (*Malus pumila*): effect on general metabolism, the phenylpropanoid pathway and cell wall enzymes. *Plant Cell Environ.* 32 1612–1631

[B30] Minas I. S. Tanou G. Belghazi M. Job D. Manganaris G. A. Molassiotis A. Vasilakakis M. (2012). Physiological and proteomic approaches to address the active role of ozone in kiwifruit post-harvest ripening. *J. Exp. Bot.* 63 2449–2464 22268155 10.1093/jxb/err418 PMC3346216

[B31] Molassiotis A. Fotopoulos V. (2011). Oxidative and nitrosative signaling in plants: two branches in the same tree? *Plant Signal. Behav.* 6 210–214 21325889 10.4161/psb.6.2.14878 PMC3121980

[B32] Molassiotis A. Tanou G. Diamantidis G. (2010). No says more than ‘YES’ to salt tolerance salt priming and systemic nitric oxide signaling in plants. *Plant Signal. Behav.* 5 209–212 20061805 10.4161/psb.5.3.10738 PMC2881262

[B33] Murata N. Iwanaga F. Maimaiti A. Imada S. Mori N. Tanaka K. Yamanaka N. (2012). Significant improvement of salt tolerance with 2-day acclimatization treatment in *Elaeagnus oxycarpa* seedlings. *Environ. Exp. Bot.* 77 170–174

[B34] Oracz K. Bouteau H. E. Farrant J. M. Cooper K. Belghazi M. Job C. Job D. Corbineau F. Bailly C. (2007). ROS production and protein oxidation as a novel mechanism for seed dormancy alleviation. *Plant J.* 50 452–465 17376157 10.1111/j.1365-313X.2007.03063.x

[B35] Rajjou L. Belghazi M. Huguet R. Robin C. Moreau A. Job C. Job D. (2006). Proteomic investigation of the effect of salicylic acid on *Arabidopsis* seed germination and establishment of early defense mechanisms. *Plant Physiol.* 141 910–923 16679420 10.1104/pp.106.082057 PMC1489900

[B36] Renaut J. Hausman J. F. Wisniewski M. E. (2006). Proteomics and low-temperature studies: bridging the gap between gene expression and metabolism. *Physiol. Plant.* 126 97–109

[B37] Slaughter A. Daniel X. Flors V. Luna E. Hohn B. Mauch-Mani B. (2012). Descendants of primed *Arabidopsis* plants exhibit resistance to biotic stress. *Plant Physiol.* 158 835–843 22209872 10.1104/pp.111.191593 PMC3271771

[B38] Stuiver C. E. E. De Kok L. J Kuiper P. J. C. (1992). Freezing tolerance and biochemical changes in wheat shoots as affected by H_2_S fumigation. *Plant Physiol. Biochem.* 30 47–55

[B39] Tanou G. Filippou P. Belghazi M. Diamantidis G. Fotopoulos V. Molassiotis A. (2012). Oxidative and nitrosative-based signaling and associated post-translational modifications orchestrate the acclimation of citrus plants to salinity stress. *Plant J.* 10.1111/j.1365-313X.2012.05100.x [Epub ahead of print]. 22780834

[B40] Tanou G. Job C. Rajjou L. Arc E. Belghazi M. Diamantidis G. Molassiotis A. Job D. (2009). Proteomics reveals the overlapping roles of hydrogen peroxide and nitric oxide in the acclimation of citrus plants to salinity. *Plant J.* 60 795–804 19682288 10.1111/j.1365-313X.2009.04000.x

[B41] Ton J. Jakab G. Toquin V. Flors V. Iavicoli A. Maeder M. Metraux J. P. Mauch-Mani B. (2005). Dissecting the beta-aminobutyric acid-induced priming phenomenon in *Arabidopsis*. *Plant Cell* 17 987–999 15722464 10.1105/tpc.104.029728 PMC1069713

[B42] Tsai C. Singh P. Chen C. Thomas J. Weber J. Mauch-Mani B. Zimmerli L. (2011). Priming for enhanced defence responses by specific inhibition of the *Arabidopsis* response to coronatine. *Plant J.* 65 469–479 21265899 10.1111/j.1365-313X.2010.04436.x

[B43] Uchida A. Jagendorf A. T. Hibino T. Takabe T. Takabe T. (2002). Effects of hydrogen peroxide and nitric oxide on both salt and heat stress tolerance in rice. *Plant Sci.* 163 515–523

[B44] Umezawa T. Shimizu K. Kato M. Ueda T. (2000). Enhancement of salt tolerance in soybean with NaCl pretreatment. *Physiol. Plant.* 110 59–63

[B45] Yacoubi R. Job C. Belghazi M. Chaibi W. Job D. (2011). Toward characterizing seed vigor in alfalfa through proteomic analysis of germination and priming. *J. Proteome Res.* 10 3891–3903 21755932 10.1021/pr101274f

[B46] Yang J. Zhang J. Liu K. Wang Z. Liu L. (2007). Involvement of polyamines in the drought resistance of rice. *J. Exp. Bot.* 58 1545–1555 17332417 10.1093/jxb/erm032

[B47] Zimmerli L. Hou B. Tsai C. Jakab G. Mauch-Mani B. Somerville S. (2008). The xenobiotic β-aminobutyric acid enhances *Arabidopsis* thermotolerance. *Plant J.* 53 144–156 18047473 10.1111/j.1365-313X.2007.03343.x

